# Supplementary material for: A Meta-Analysis of Influencing Factors on the Activity of BiVO4-Based Photocatalysts
Source: Nanomaterials (Basel). 2023 Aug 16;13(16):2352. doi: 10.3390/nano13162352 (PMC10458677; doi:10.3390/nano13162352)
Supplement: Supplementary file 1 [file nanomaterials-13-02352-s001.zip › nanomaterials-2554911-supplementary.pdf]

Supplementary information

A Meta-Analysis of Influencing Factors on the Activity of $BiVO_4$-Based Photocatalysts

Ruijie Che [1,2,3,†], Yining Zhu [1,3,†], Biyang Tu [1,†], Jiahe Miao [1], Zhongtian Dong [3], Mengdi Liu [1], Yupeng Wang [4], Jining Li [1,3,*], Shuoping Chen [2,*] and Fenghe Wang [3,*]

[1] School of Environment, Nanjing Normal University, Nanjing 210023, China; 2120210253@glut.edu.cn (R.C.); 222502023@njnu.edu.cn (Y.Z.); 212501004@njnu.edu.cn (B.T.); jh963266@dal.ca (J.M.); 222512031@njnu.edu.cn (M.L.)

[2] School of Materials Science and Engineering, Guilin University of Technology, Guilin 541010, China

[3] Key Laboratory for Soft Chemistry and Functional Materials of Ministry of Education, Nanjing University of Science and Technology, Nanjing 210094, China; dztnoone@foxmail.com

[4] School of Pharmacy, Nanjing Technology University, Nanjing 211816, China; 202261109033@njtech.edu.cn

[*] Correspondence: lijining@njnu.edu.cn (J.L.); 2012014@glut.edu.cn (S.C.); wangfenghe@njnu.edu.cn (F.W.)

[†] These authors contributed equally to this work.

Table S1 Effects of coupling type, time, temperature, and preparation pH on the amount of BET effect in $BiVO_4$-based composites

	Variable	Group	Sample size (n)	SMD	95% CI (SMD)	
					Lower	Upper
Preparation conditions	Coupling Type	Metal	3	0.92	0.14	1.7
		Oxide	10	6.22	5.8	6.64
		Sulfide	3	0.39	−0.61	1.39
		Carbon Materials	4	5.45	4	6.91
		Heterojunctions	2	5.98	4.46	7.51
		Nanomaterials	4	12.23	11.65	12.8
	Time	1–6 h	9	9.8	9.1	10.49
		6–24 h	7	3.45	3.2	3.7
		>24 h	4	0.67	−0.04	1.37
	Temperature	Low (≤100	9	1.88	1.5	2.25
		Medium (100~200	9	5.87	5.56	6.18
		High (≥200	4	5.06	4.49	5.63
	Degradation pH	Acidic conditions	4	12.06	11.4	12.73
		Neutral	4	4.87	4.24	5.51
		Alkaline conditions	5	3.3	2.5	4.1

Table S2 Types of coupling and their loading of $BiVO_4$-based composites in various literatures

References	Coupling type	Loading capacity (wt%)
[1]	Metal	7
[2]		0.5
[3]	Oxide	3
[4]		5
[5]		42.86 (molar ratio)
[6]		2.5

[7]		60
[8]		-
[9]		3
[10]		
[11]		10
[12]		1.5
[13]		0.7 (molar ratio)
[14]	Sulfide	1:2 (S/V)
[15]		4
[16]		3
[17]	Carbon Materials	10
[18]		12
[19]		20
[20]	Heterojunctions	1.5
[21]		2
[22]		0.02
[23]	Nanomaterials	-
[24]		1
[25]		-

Table S3 Effects of preparation and degradation conditions on the kinetic constant effector quantities of $BiVO_4$-based composites

	Variable	Group	Sample size (n)	SMD	95% CI (SMD)	
					Lower	Upper
Preparation conditions	Coupling Type	Metal	3	–0.81	–1.46	–0.15
		Oxide	10	0.58	–0.1	1.26
		Sulfide	3	0.43	–2.89	3.75
		Carbon Materials	4	2.4	0.48	4.32
		Heterojunctions	2	0	–2.4	2.4
		Nanomaterials	4	0.26	–0.94	1.47
	Time	1–6 h	9	0.57	–0.14	1.27
		6–24 h	7	0.64	–0.03	1.32
		>24 h	4	0.83	–1.46	3.12
	Temperature	Low (≤100	9	0.59	–0.6	1.78

		Medium (100~200	9	0.76	0.06	1.45
		High (≥200	4	0.36	–0.98	1.7
	Degradation pH	Acidic conditions	4	1.62	–0.64	3.88
		Neutral	4	1.71	–0.02	3.44
		Alkaline conditions	5	1.61	0.04	3.18
Degradation condition	Pollution concentration (mg/L)	<20	13	0.46	–0.45	1.37
		20–200	7	0.97	–1.17	3.11
		>200	2	0.05	–0.9	1.01
	Photocatalyst dosage (g/L)	<0.5	6	0.31	–0.79	1.41
		0.5–1	14	0.77	0.23	1.32
		>1	4	0.49	–0.84	1.82
	Solution pH	Acidic	8	0.27	–1.95	2.49
		Neutral	3	0.46	–0.28	1.21
		Alkaline	4	0.27	–1.95	2.49

Table S4 Effects of different preparation and degradation conditions on the photocatalytic performance of $BiVO_4$-based composites for the degradation of organic pollutants

	**Variable**	**Group**	**Sample size (n)**	**SMD**	**95% CI (SMD)**	
					**Lower**	**Upper**
Preparation conditions	Coupling Type	Metal	3	11.51	10.67	12.34
		Oxide	10	5.41	5.17	5.64
		Sulfide	3	11.28	10.42	12.14
		Carbon Materials	4	9.82	9.22	10.42
		Heterojunctions	1	11.92	10.86	12.97
		Nanomaterials	3	14.29	13.33	15.25
	Time	1–6 h	8	9.6	9.28	9.92

		6–24 h	6	8.24	7.93	8.55
		>24 h	4	14.61	13.71	15.5
	Temperature	Low (≤100	8	8.62	8.24	9
		Medium (100~200	8	9.01	8.7	9.31
		High (≥200	4	13.01	12.3	13.72
	Degradation pH	Acidic conditions	4	8.87	8.33	9.41
		Neutral	2	13.01	11.83	14.19
		Alkaline conditions	5	10.92	10.33	11.52
Degradation condition	Pollution concentration (mg/L)	<20	11	10.04	9.67	10.4
		20–200	7	15.65	14.94	16.35
		>200	2	13.89	12.55	15.23
	Photocatalyst dosage (g/L)	<0.5	6	6.37	5.99	6.75
		0.5–1	13	7.5	7.23	7.76
		>1	4	5.69	5.32	6.06
	Solution pH	Acidic	8	13.31	12.7	13.93
		Neutral	3	12.74	11.86	13.62
		Alkaline	3	3.41	3.11	3.71

Table S5 Effects of different preparation and degradation conditions on the photocatalytic performance of $BiVO_4$-based composites for the reduction of inorganic pollutants

	**Variable**	**Group**	**Sample size (n)**	**SMD**	**95% CI (SMD)**	
					**Lower**	**Upper**
Preparation conditions	Coupling Type	Metal	0	0	0	0
		Oxide	0	0	0	0
		Sulfide	0	0	0	0
		Carbon Materials	0	0	0	0

		Heterojunctions	1	9.2	8.06	10.34
		Nanomaterials	1	10.16	8.96	11.36
	Time	1–6 h	1	10.13	8.88	11.37
		6–24 h	1	11.11	9.81	12.42
		>24 h	0	0	0	0
	Temperature	Low (≤100	1	8.18	7.15	9.2
		Medium (100~200	1	11.69	10.32	13.06
		High (≥200	0	0	0	0
	Degradation pH	Acidic conditions	0	0	0	0
		Neutral	2	9.04	8.27	9.81
		Alkaline conditions	0	0	0	0
Degradation condition	Pollution concentration (mg/L)	<20	2	10.64	9.74	11.54
		20–200	0	0	0	0
		>200	0	0	0	0
	Photocatalyst dosage (g/L)	<0.5	0	0	0	0
		0.5–1	1	5.63	5.11	6.16
		>1	0	0	0	0
	Solution pH	Acidic	0	0	0	0
		Neutral	0	0	0	0
		Alkaline	1	8.12	7.14	9.1

References

1. Lebedev, A.; Anariba, F.; Li, X.; Wu, P. Rational Design of Visible-Light-Sensitive Ag-BiVO4 Oxides by Matching Redox Potentials of Catalyst, Dyes, and Reactive Oxygen Species towards More Efficient Photocatalytic Degradation. *Journal of Environmental Chemical Engineering* **2020**, *8*, 103748, doi:10.1016/j.jece.2020.103748.
2. Oladipo, A.A.; Mustafa, F.S. Bismuth-Based Nanostructured Photocatalysts for the Remediation of Antibiotics and Organic Dyes. *Beilstein J. Nanotechnol.* **2023**, *14*, 291–321, doi:10.3762/bjnano.14.26.
3. Fatima, U.; Tahir, M.B.; Gouadria, S.; Khalid, N.R.; Nawaz, T.; Sagir, M.; Siddeeg, S.M.; Alrobei, H.; Alzaid, M. Synthesis of Ternary Photocatalysts BiVO4/Ag/Black Phosphorene for the Degradation of Dyes and Pharmaceuticals. *Appl Nanosci* **2023**, *13*, 5501–5507, doi:10.1007/s13204-023-02762-0.
4. Liaqat, M.; Khalid, N.R.; Tahir, M.B.; Znaidia, S.; Alrobei, H.; Alzaid, M. Visible Light Induced Photocatalytic Activity of MnO2/BiVO4 for the Degradation of Organic Dye and Tetracycline. *Ceramics International* **2023**, *49*, 10455–10461, doi:10.1016/j.ceramint.2022.11.229.
5. Bano, K.; Mittal, S.K.; Singh, P.P.; Kaushal, S. Sunlight Driven Photocatalytic Degradation of Organic Pollutants Using a $MnV_2O_6/BiVO_4$ Heterojunction: Mechanistic Perception and Degradation Pathways. *Nanoscale Adv.* **2021**, *3*, 6446–6458, doi:10.1039/D1NA00499A.
6. Yuan, Q.; Chen, L.; Xiong, M.; He, J.; Luo, S.-L.; Au, C.-T.; Yin, S.-F. Cu2O/BiVO4 Heterostructures: Synthesis and Application in Simultaneous Photocatalytic Oxidation of Organic Dyes and Reduction of Cr(VI) under Visible Light. *Chemical Engineering Journal* **2014**, *255*, 394–402, doi:10.1016/j.cej.2014.06.031.
7. Zhu, P.; Zhang, S.; Liu, R.; Luo, D.; Yao, H.; Zhu, T.; Bai, X. Investigation of an Enhanced Z-Scheme Magnetic Recyclable BiVO4/GO/CoFe2O4 Photocatalyst with Visible-Light-Driven for Highly Efficient Degradation of Antibiotics. *Journal of Solid State Chemistry* **2022**, *314*, 123379, doi:10.1016/j.jssc.2022.123379.
8. Soltani, T.; Tayyebi, A.; Lee, B.-K. Photolysis and Photocatalysis of Tetracycline by Sonochemically Heterojunctioned BiVO4/Reduced Graphene Oxide under Visible-Light Irradiation. *Journal of Environmental Management* **2019**, *232*, 713–721, doi:10.1016/j.jenvman.2018.11.133.
9. Zheng, X.; Li, Y.; Peng, H.; Huang, Z.; Wang, H.; Wen, J. Efficient Solar-Light Photodegradation of Tetracycline Hydrochloride Using BiVO4/MoO3 Composites. *Colloids and Surfaces A: Physicochemical and Engineering Aspects* **2021**, *621*, 126599, doi:10.1016/j.colsurfa.2021.126599.
10. Zheng, Y.; Cao, L.; Xing, G.; Bai, Z.; Huang, J.; Zhang, Z. Microscale Flower-like Magnesium Oxide for Highly Efficient Photocatalytic Degradation of Organic Dyes in Aqueous Solution. *RSC Adv.* **2019**, *9*, 7338–7348, doi:10.1039/C8RA10385B.
11. Wei, L.; Shi, D.; Qi, Y.; Zhang, Y. Synthetic Mechanism of $UiO\text{-}66\text{-}NH_2/BiVO_4/BiOBr$ Spherical and Lamellar Dual Z-scheme Heterojunction and Efficient Photocatalytic Degradation of Tetracycline Under Visible Light. *ChemistrySelect* **2022**, *7*, doi:10.1002/slct.202103742.
12. Tahir, M.B.; Iqbal, T.; Kiran, H.; Hasan, A. Insighting Role of Reduced Graphene Oxide in

BiVO 4 Nanoparticles for Improved Photocatalytic Hydrogen Evolution and Dyes Degradation. *Int J Energy Res* **2019**, *43*, 2410–2417, doi:10.1002/er.4443.

13. Li, Y.; Li, X.; Wang, X.-T.; Jian, L.-J.; Abdallah, N.I.M.; Dong, X.-F.; Wang, C.-W. P-n Heterostructured Design of Decahedral NiS/BiVO4 with Efficient Charge Separation for Enhanced Photodegradation of Organic Dyes. *Colloids and Surfaces A: Physicochemical and Engineering Aspects* **2021**, *608*, 125565, doi:10.1016/j.colsurfa.2020.125565.
14. Liang, Q.; Ploychompoo, S.; Chen, J.; Zhou, T.; Luo, H. Simultaneous Cr(VI) Reduction and Bisphenol A Degradation by a 3D Z-Scheme Bi2S3-BiVO4 Graphene Aerogel under Visible Light. *Chemical Engineering Journal* **2020**, *384*, 123256, doi:10.1016/j.cej.2019.123256.
15. Yan, X.; Wang, B.; Zhao, J.; Liu, G.; Ji, M.; Zhang, X.; Chu, P.K.; Li, H.; Xia, J. Hierarchical Columnar ZnIn2S4/BiVO4 Z-Scheme Heterojunctions with Carrier Highway Boost Photocatalytic Mineralization of Antibiotics. *Chemical Engineering Journal* **2023**, *452*, 139271, doi:10.1016/j.cej.2022.139271.
16. Le, S.; Li, W.; Wang, Y.; Jiang, X.; Yang, X.; Wang, X. Carbon Dots Sensitized 2D-2D Heterojunction of BiVO4/Bi3TaO7 for Visible Light Photocatalytic Removal towards the Broad-Spectrum Antibiotics. *Journal of Hazardous Materials* **2019**, *376*, 1–11, doi:10.1016/j.jhazmat.2019.04.088.
17. Zhao, J.; Yan, J.; Jia, H.; Zhong, S.; Zhang, X.; Xu, L. BiVO 4 /g-C 3 N 4 Composite Visible-Light Photocatalyst for Effective Elimination of Aqueous Organic Pollutants. *Journal of Molecular Catalysis A: Chemical* **2016**, *424*, 162–170, doi:10.1016/j.molcata.2016.08.025.
18. Huang, L.; Liu, H.; Wang, Y.; Zhang, T.C.; Yuan, S. Construction of Ternary Bi2O3/Biochar/g-C3N4 Heterojunction to Accelerate Photoinduced Carrier Separation for Enhanced Tetracycline Photodegradation. *Applied Surface Science* **2023**, *616*, 156509, doi:10.1016/j.apsusc.2023.156509.
19. Gu, J.; Ban, C.; Meng, J.; Li, Q.; Long, X.; Zhou, X.; Liu, N.; Li, Z. Construction of Dual Z-Scheme UNiMOF/BiVO4/S-C3N4 Photocatalyst for Visible-Light Photocatalytic Tetracycline Degradation and Cr(VI) Reduction. *Applied Surface Science* **2023**, *611*, 155575, doi:10.1016/j.apsusc.2022.155575.
20. Zhang, X.; Ma, Y.; Xi, L.; Zhu, G.; Li, X.; Shi, D.; Fan, J. Highly Efficient Photocatalytic Removal of Multiple Refractory Organic Pollutants by BiVO4/CH3COO(BiO) Heterostructured Nanocomposite. *Science of The Total Environment* **2019**, *647*, 245–254, doi:10.1016/j.scitotenv.2018.07.450.
21. Xu, G.; Du, M.; Zhang, J.; Li, T.; Guan, Y.; Guo, C. Facile Fabrication of Magnetically Recyclable Fe3O4/BiVO4/CuS Heterojunction Photocatalyst for Boosting Simultaneous Cr(VI) Reduction and Methylene Blue Degradation under Visible Light. *Journal of Alloys and Compounds* **2022**, *895*, 162631, doi:10.1016/j.jallcom.2021.162631.
22. Mosleh, S.; Rahimi, M.R.; Ghaedi, M.; Dashtian, K. HKUST-1-MOF–BiVO 4 Hybrid as a New Sonophotocatalyst for Simultaneous Degradation of Disulfine Blue and Rose Bengal Dyes: Optimization and Statistical Modelling. *RSC Adv.* **2016**, *6*, 61516–61527, doi:10.1039/C6RA13837C.
23. Shang, M.; Wang, W.; Zhou, L.; Sun, S.; Yin, W. Nanosized BiVO4 with High Visible-Light-Induced Photocatalytic Activity: Ultrasonic-Assisted Synthesis and Protective Effect of Surfactant. *Journal of Hazardous Materials* **2009**, *172*, 338–344, doi:10.1016/j.jhazmat.2009.07.017.

24. Shahzad, K.; Tahir, M.B.; Sagir, M.; Kabli, M.R. Role of CuCo2S4 in Z-Scheme MoSe2/BiVO4 Composite for Efficient Photocatalytic Reduction of Heavy Metals. *Ceramics International* **2019**, *45*, 23225–23232, doi:10.1016/j.ceramint.2019.08.018.
25. Sajid, M.Munir.; Amin, N.; Shad, N.A.; Khan, S.B.; Javed, Y.; Zhang, Z. Hydrothermal Fabrication of Monoclinic Bismuth Vanadate (m-BiVO4) Nanoparticles for Photocatalytic Degradation of Toxic Organic Dyes. *Materials Science and Engineering: B* **2019**, *242*, 83–89, doi:10.1016/j.mseb.2019.03.012.
